# Change in cephalocaudal tumor cavity diameter after transsphenoidal surgery is a predictor of diabetes insipidus in pituitary adenoma

**DOI:** 10.1186/s40001-022-00700-4

**Published:** 2022-05-25

**Authors:** Kunzhe Lin, Kaichun Fan, Shuwen Mu, Shousen Wang

**Affiliations:** 1 Department of Neurosurgery, Affiliated Fuzhou First Hospital of Fujian Medical University, Fuzhou, 350009 China; 2 Fuzong Clinical Medical College of Fujian Medical University, Fuzhou, 350025 China; 3 Department of Gastroenterology and Hepatology, The First Medical Center, Chinese PLA General Hospital, Beijing, 100853 China; 4 Department of Neurosurgery, 900th Hospital, Fuzhou, 350025 China

**Keywords:** Diabetes insipidus, Transsphenoidal surgery, Pituitary adenoma, Cephalocaudal tumor cavity, Multivariate

## Abstract

**Objective:**

To assess the factors influencing the development of diabetes insipidus after transsphenoidal surgery for pituitary adenomas.

**Methods:**

We retrospectively analyzed the clinical data of patients with pituitary adenoma who underwent transsphenoidal surgery. The pituitary gland was assessed using a 3.0 T magnetic resonance imaging, and the predictors of postoperative diabetes insipidus were determined through univariate and multivariate analyses.

**Results:**

A total of 212 eligible patients with pituitary adenomas were included; 82 (38.7%) cases developed postoperative diabetes insipidus while 130 cases (61.3%) did not. Diabetes insipidus was transient in 80 (37.7%) patients and permanent in 2 (0.9%) patients. The results of logistic regression analyses showed that the change in cephalocaudal tumor cavity diameter after transsphenoidal surgery was associated with the occurrence of postoperative diabetes insipidus.

**Conclusions:**

Change in cephalocaudal tumor cavity diameter after transsphenoidal surgery may play an important role in predicting diabetes insipidus onset in patients with a pituitary adenoma.

## Introduction

Diabetes insipidus (DI) is common complication after transsphenoidal surgery (TSS) for pituitary adenoma, and its incidence fluctuates between 16.6 and 45.8% [1–6]. DI is caused by surgical damage to the hypothalamus–neurohypophyseal axis, which in turn causes a decrease in ADH secretion [2, 6, 7]. It usually manifests as polydipsia, polyuria, thirst, and low specific gravity urine [8]. Untreated patients can develop dehydration and hypernatremia, which can cause mental changes, epilepsy, hypotension, coma, and renal failure [9, 10]. Therefore, timely prediction and treatment of patients developing DI post-surgery can improve patient outcomes, reduce the length of hospital stay, and reduce the cost of treatment.

Though several factors predicting the occurrence of postoperative DI, including cephalocaudal tumor diameter, age, degree of deformation of the third ventricle and hypothalamus, total tumoral resection, intraoperative cerebrospinal fluid (CSF) leak, and microadenoma [2–4, 7] have been previously reported, a definitive consensus is yet to be reached. In addition, these studies did not provide a possible basis for the damage to the hypothalamus, pituitary stalk, and posterior pituitary, nor did they control for these factors to reduce the probability of postoperative DI.

Considering the challenges involved in evaluating the damage to the hypothalamus–neurohypophyseal system during surgery, we intended to explore a novel mechanism to assess intraoperative injury by magnetic resonance imaging (MRI) and explore its use in predicting DI after TSS.

## Materials and methods

### Patients

A retrospective analysis was conducted on the clinical data of patients admitted to the Department of Neurosurgery with pituitary adenoma, who underwent TSS between January 2017 and January 2021. This study protocol was reviewed and approved by hospital. All study participants provided informed consent. All data were anonymized to protect patient confidentiality.

We included patients who (1) underwent microscopic TSS for pituitary adenomas by the same physician with more than 20 years of experience in pituitary surgery; (2) had a tumor that grew upward beyond the plane of the original diaphragma sellae; (3) underwent repeated MRI examination in the 2–3 days following the surgery, and (4) underwent pituitary surgery for the first time. We excluded patients with (1) a preoperative history of radiotherapy, (2) a tumor that did not exceed the plane of the diaphragma sellae, and (3) pathologically confirmed pituitary adenoma with other concomitant sellar lesions.

### Data collection

All data were collected retrospectively by reviewing patients’ electronic medical records. Data on demographics, patient’s age, sex, preoperative and postoperative MRI features, surgical records, immunohistological diagnosis, electrolyte levels, urine output and time from the day of surgery until the day of discharge were investigated.

### Radiological evaluation

**Fig. 1:**
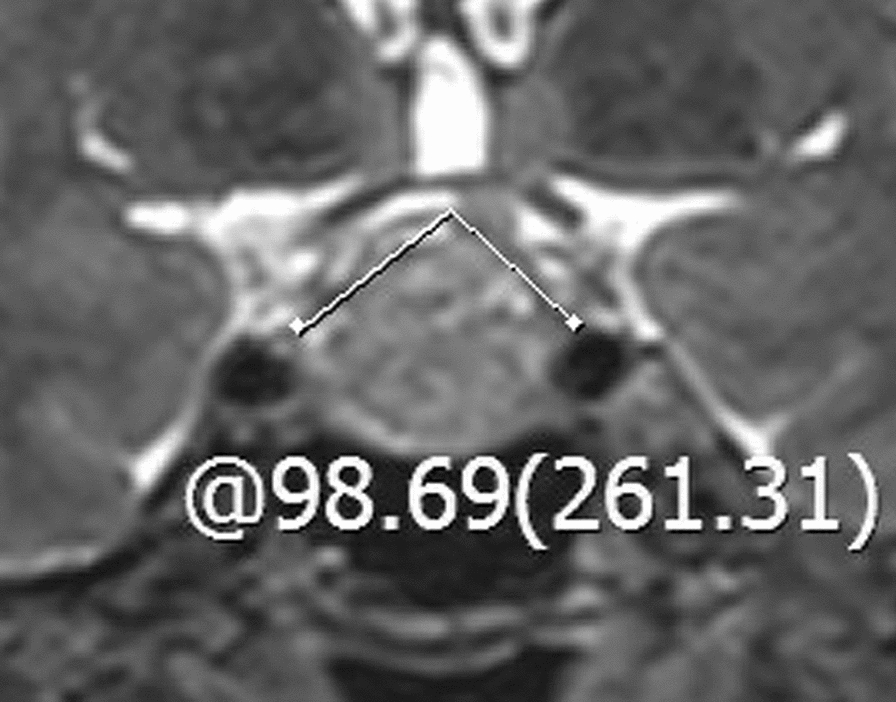
The imaginary angle drawn between the two edges of diaphragma sellae begins its elevation and the highest point of the diaphragma sellae

**Fig. 2:**
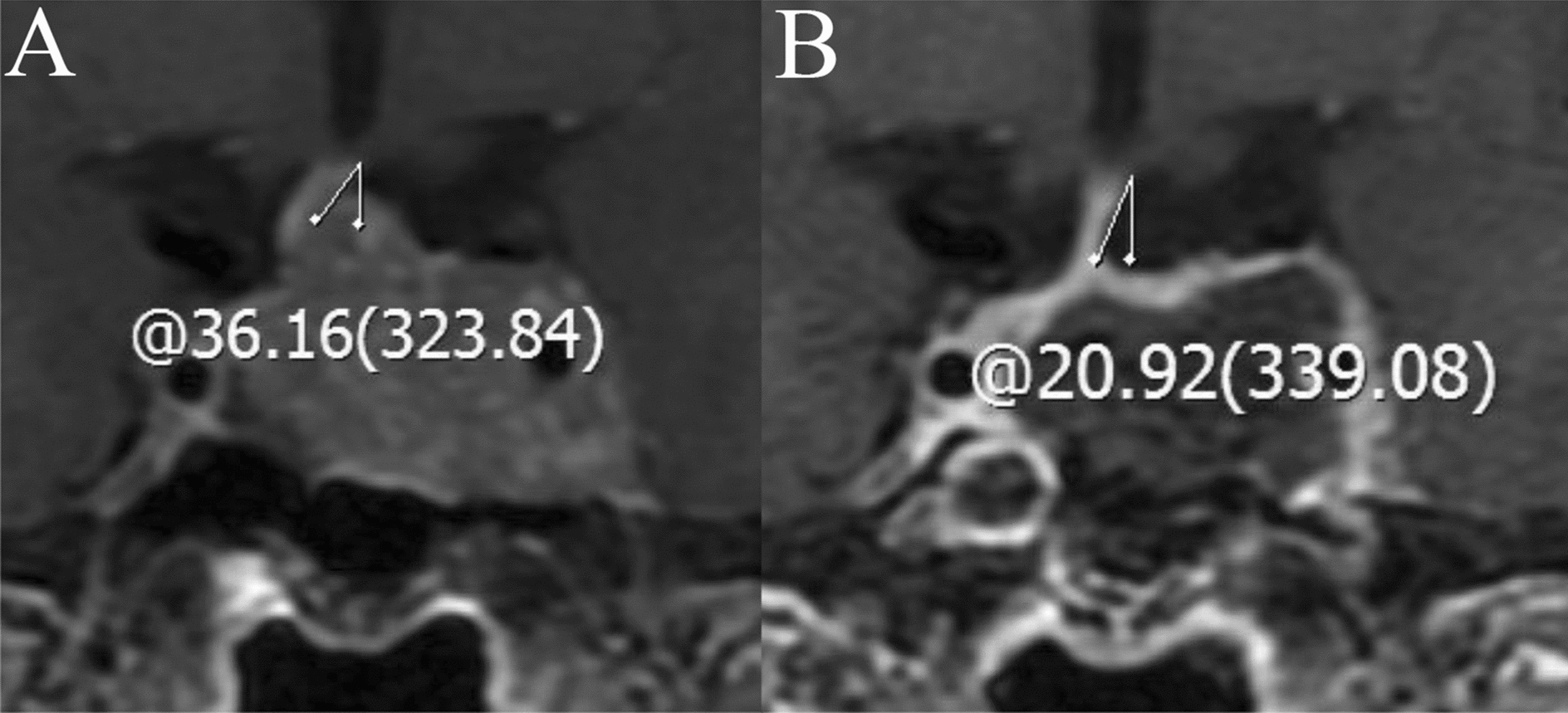
Coronal contrast-enhanced images of the difference in pituitary stalk deviation angle before and after transsphenoidal surgery. **A** Before surgery, the pituitary stalk deviates 36.16° to the right. **B** After surgery, the pituitary stalk deviation angle of this patient is 36.16° to 20.92°

**Fig. 3:**
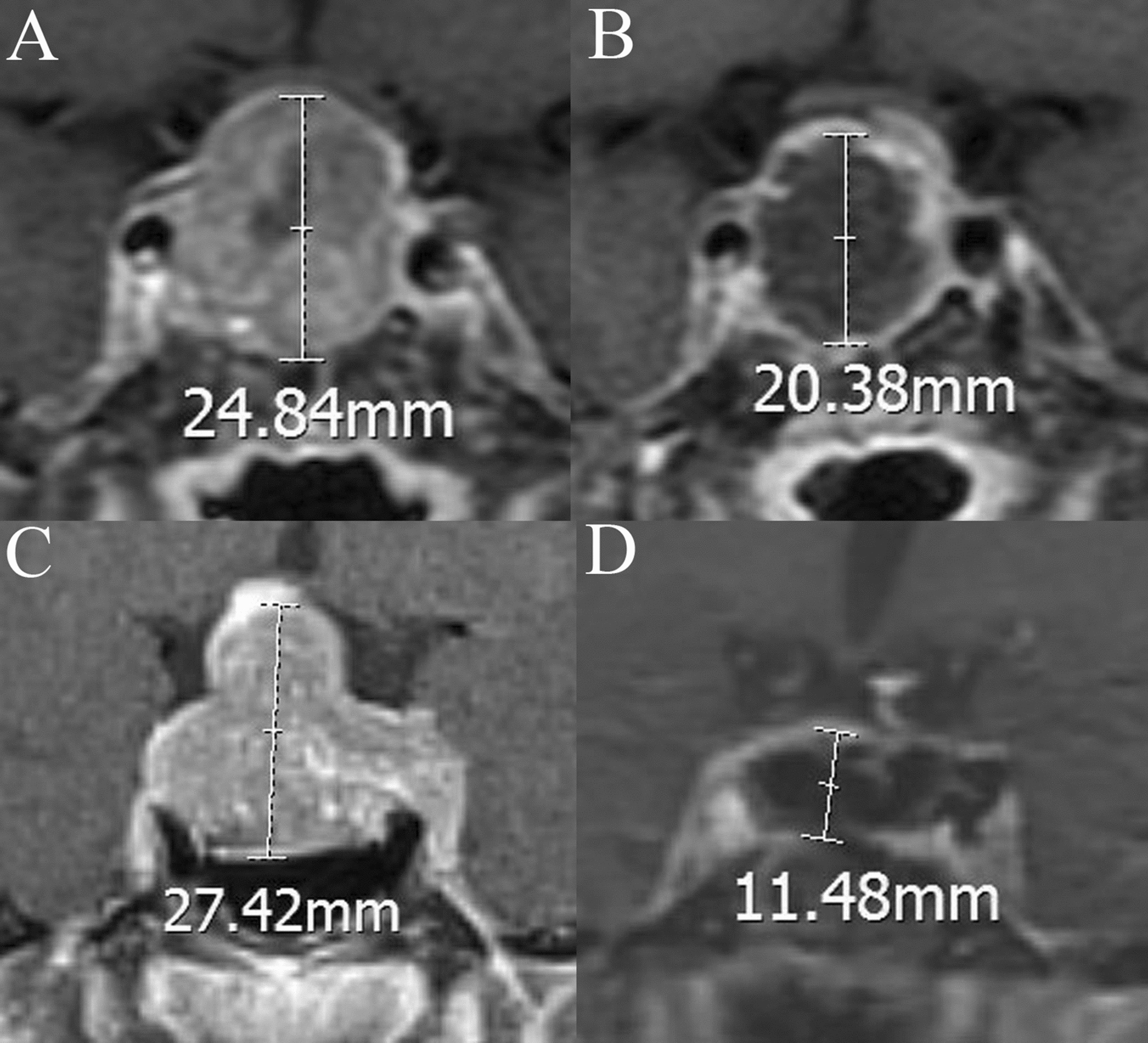
Coronal contrast-enhanced images of the change in cephalocaudal tumor cavity diameter after transsphenoidal surgery in 2 cases. **A**–**B**, **C**–**D** are from the same patient, respectively. **A** Before surgery, the cephalocaudal tumor cavity diameter is 24.84 mm. **B** After surgery, the cephalocaudal tumor cavity diameter is 20.38 mm, and the change in cephalocaudal tumor cavity diameter after transsphenoidal surgery of this patient is 24.84–20.38 mm. **C** Before surgery, the cephalocaudal tumor cavity diameter is 27.42 mm. **D** After surgery, the cephalocaudal tumor cavity diameter is 11.48 mm, and the change in cephalocaudal tumor cavity diameter after transsphenoidal surgery of this patient is 27.42–11.48 mm

The pituitary gland was scanned using a 3.0 T MRI scanner (Tim Trio; Siemens Medical Solutions, Erlangen, Germany). T1-weighted, T2-weighted, and contrast-enhanced images were evaluated to identify potential factors for postoperative DI, which included cephalocaudal tumor cavity diameter (preoperative and postoperative), tumor volume, intratumoral cysts or hematoma, posterior pituitary bright spot (PPBS) occurrence, invasiveness, pituitary stalk deviation angle (preoperative and postoperative), and the imaginary angle at the highest point of the diaphragma sellae between the two edges where the diaphragma sellae begins its elevation (Fig. 1). The angle at which the starting part of the pituitary stalk deviates from the midline was defined as the deviation angle of the pituitary stalk [11] (Fig. 2). The cephalocaudal tumor cavity diameter was measured pre- and post-operatively at the same coronal position on contrast-enhanced images, and the difference between the two was taken as the change in cephalocaudal tumor cavity diameter after TSS (Fig. 3). Tumor volume was calculated using the platform-like volume calculation formula [12]. Patients with Knosp Grades 3 or 4 were defined as having cavernous sinus invasion [13]. The extent of tumor resection was classified as gross total resection when there was no evidence of residual adenoma; subtotal resection when residual adenoma < 20%; and partial resection when residual adenoma < 50% [14]. The magnetic resonance images were independently evaluated and measured by a neurosurgeon and a neuroradiologist. The kappa (*κ*) index was used to evaluate interobserver and intraobserver concordance.

### Diagnostic criteria for DI

The diagnostic criteria for DI were as follows [15, 16]: (1) hypotonic polyuria (urine output > 3 L/24 h, urine specific gravity < 1.005, urine osmolality < 300 mOsm/kg); (2) normal or elevated blood sodium; (3) reduced urine output after treatment with desmopressin; and (4) exclusion of other causes of polyuria.

### Statistical analysis

All statistical analyses were performed using IBM SPSS Statistics for Windows, Version 20 (IBM Corp., Armonk, NY, USA). The clinical data were expressed as mean and standard deviation, interquartile range, and frequency and percentage, as appropriate. Independent samples *t*-test, Wilcoxon rank-sum test, Fisher’s exact test, Chi-square test, and logistic regression were performed. *P* < 0.05 was considered to be statistically significant.

## Results

During the study period, a total of 323 cases underwent TSS. Ultimately, 212 eligible patients were included, among whom 82 (38.7%) developed postoperative DI and 130 cases (61.3%) did not. DI was transient in 80 (37.7%) patients and permanent in 2 (0.9%) patients. The median onset of DI was on postoperative day 1, and the median duration of transient DI was 3.5 days.

Table 1 summarizes the comparisons of the data between the DI and non-DI groups. The preoperative cephalocaudal tumor cavity diameter of the non-DI group was significantly lower than that of the DI group (*P* = 0.016). The change in cephalocaudal tumor cavity diameter after TSS was significantly larger in patients with DI compared to those without (*P* < 0.001). The difference between preoperative and postoperative pituitary stalk deviation angles was greater in patients with DI (*P* = 0.003). There were no significant differences between the DI and non-DI groups in terms of age, sex, immunohistochemical types, PPBS occurrence, tumor cystic degeneration or hemorrhage, intraoperative CSF, extent of tumor resection, and length of hospital stay after surgery.

**Table 1:** Univariate analysis of postoperative DI

Factors	Non-postoperative DI (*n* = 130)	Postoperative DI (*n* = 82)	*P *value
Age (year)	48 (37, 59)	51 (40, 63)	0.540
Sex			
Wen	65 (30.7%)	50 (23.6%)	0.118
Women	65 (30.7%)	32 (15.1%)	
Preoperative cephalocaudal tumor cavity diameter (mm)	23.0 (18.7, 28.7)	26.4 (20.9, 33.9)	0.016
Tumor size (cm^3^)	4.1 (2.4, 6.6)	4.9 (2.8, 8.1)	0.104
Tumor size classification			
Macroadenoma	112 (52.8%)	37 (17.5%)	< 0.001
Giant pituitary adenoma	18 (8.5%)	45 (21.2%)	
Pathological tumor type			
Gonadotropinoma	39 (18.4%)	35 (16.5%)	0.536
Null-cell adenoma	34 (16.0%)	20 (9.4%)	
PRL cell adenoma	19 (8.9%)	7 (3.3%)	
GH cell adenoma	11 (5.2%)	4 (1.9%)	
ACTH cell adenoma	15 (7.1%)	8 (3.8%)	
Plurihormonal	11 (5.2%)	7 (3.3%)	
Thyroid cell adenoma	1 (0.5%)	1 (0.5%)	
Intratumoral cysts or hematoma			
Yes	48 (22.6%)	40 (18.9%)	0.088
No	82 (38.7%)	42 (19.8%)	
PPBS occurrence			
Positive	116 (54.7%)	71 (33.5%)	0.561
Negative	14 (6.6%)	11 (5.2%)	
Invasiveness			
Yes	20 (9.4%)	12 (5.7%)	0.882
No	110 (51.9%)	70 (33.0%)	
The imaginary angle (°)	91.0 (75.7, 105.3)	86.0 (68.75, 97.3)	0.069
Postoperative cephalocaudal Tumor cavity diameter (mm)	15.3 (12.0, 19.1)	14.6 (10.0, 17.7)	0.08
Change in cephalocaudal tumor cavity diameter after TSS (mm)	7.17 (2.8, 11.9)	10.6 (5.9, 17.1)	< 0.001
Preoperative pituitary stalk deviation angle (°)	40.5 (29.5, 51.0)	45.5 (29.7, 55.0)	0.061
Postoperative pituitary stalk deviation angle (°)	29.0 (16.5, 40.5)	29.0 (16.0, 38.5)	0.612
Difference in pituitary stalk deviation angle (°)	3 (0, 17.2)	11.5 (0, 22)	0.003
Extent of tumor resection			
Total resection	103 (48.6%)	70 (33.0%)	0.515
Subtotal resection	17 (8.0%)	7 (3.3%)	
Partial resection	10 (4.7%)	5 (2.4%)	
Intraoperative cerebrospinal fluid leaks			
Yes	22 (10.3%)	16 (7.5%)	0.632
No	108 (50.9%)	66 (31.3%)	
Length of stay after surgery	7 (6, 8)	7 (6, 9)	0.229

Data were expressed as means (standard deviations), medians (interquartile ranges) or numbers (proportions)

*DI *diabetes insipidus, *PPBS* posterior pituitary bright spot, *TSS* transsphenoidal surgery, The imaginary angle the imaginary angle drawn between the two edges of diaphragm sellae begins its elevation and the highest point of the diaphragm sellae

The results of logistic regression analyses are shown in Table 2. The change in cephalocaudal tumor cavity diameter after TSS (odds ratio = 1.080, 95% confidence interval: 1.005–1.160; *P* = 0.037) was found to be significantly associated with postoperative DI.

**Table 2:** Logistic regression analysis of risk of postoperative DI onset

Factors	OR	95% CI	*P* value
Change in cephalocaudal tumor cavity diameter after TSS	1.080	1.005, 1.160	0.037
Difference in pituitary stalk deviation angle	1.017	0.992, 1.042	0.192
Preoperative cephalocaudal tumor cavity diameter	0.979	0.924, 1.036	0.457
Tumor size classification	1.537	0.535, 4.412	0.424

*DI* diabetes insipidus; *OR* odds ratio, *CI* confidence interval, *TSS* transsphenoidal surgery

## Discussion

Our study evaluated the predictive factors of DI after TSS in pituitary adenoma. For the first time, we found that the change in cephalocaudal tumor cavity diameter after TSS was an independent risk factor for DI.

The incidence rates of DI after TSS are 16.6% [1], 18.3% [3], 26% [17], 42.6% [18], and 45.8% [4] as reported by different studies. Thus, the incidence of DI varies among centers. On the one hand, the occurrence of DI depends on the damage to the hypothalamic–neurohypophyseal system, and on the other, it may be explained by the wide range of criteria used to establish a diagnosis of DI in the literature [16]. The incidence of DI in this study was 38.7%, and more cases were that of transient DI.

Oh et al. showed that the cephalocaudal tumor diameter could predict the occurrence of DI after TSS [4]. They speculated that when the cephalocaudal tumor diameter was large, the tumor would affect the release of diet-related hormones in the hypothalamus leading to obesity. This could further result in narrowing of the nasal cavity, increasing the difficulty of surgery, and therefore increasing the risk of injury to the pituitary stalk and posterior pituitary lobe. In our study, the cephalocaudal tumor diameter before surgery in the DI group was larger than that in the non-DI group. However, logistic regression analysis did not find evidence that cephalocaudal tumor diameter could predict DI after TSS. We found that the change in cephalocaudal tumor cavity diameter after TSS was an independent risk factor for DI.

When the tumor grows upwards, the pituitary tissue and diaphragma sellae can be pushed [19]. In addition, the pituitary stalk may show chronic distortion changes [3]. On contrast-enhanced MRI images, compressed and flattened normal pituitary tissue was observed above the tumor. Therefore, the change in cephalocaudal tumor cavity diameter reflects the downward displacement of the diaphragma sellae, pituitary stalk, and normal pituitary tissue after tumor resection. With the gradual removal of the tumor during the operation, different degrees of subsidence of the diaphragma sellae can be seen. These sinking structures consist of one or more of the following: the diaphragm itself, arachnoid membrane, tumor pseudocapsule, and flat normal pituitary tissue. Therefore, the above structure refers to the diaphragm sellae from a surgical perspective, not an anatomical perspective [20]. The greater the change in cephalocaudal tumor cavity diameter, more obviously is the pituitary stalk displaced and pulled, and therefore, the more likely it is to be damaged leading to DI. We believe that the diaphragma sellae subsidence occurred intraoperatively after tumor resection, an acute process. Meanwhile, after the tumor cavity was packed with gelatin sponge, the diaphragma sellae was lifted upwards, and this postoperative change in cephalocaudal tumor cavity diameter was a chronic process. We followed up for patients 3 months postoperatively and repeating the MRI, which revealed further subsidence of the diaphragma sellae.

The pituitary stalk and posterior lobe of pituitary gland may change in position and shape when the tumor grows slowly to the suprasellar region for a long time, and the pituitary stalk may be distorted [21, 22]. In this study, the difference in the pituitary stalk deviation angle before and after surgery in the DI group was greater than that in the non-DI group. This indicated that the position of the pituitary stalk changed significantly in DI group. Sinking of the diaphragma sellae during surgery may have potentially pulled and changed the shape and position of the pituitary stalk, resulting in injury to the hypothalamic–neurohypophysis system and triggering the occurrence of DI after TSS. Thus, we propose a new mechanism of injury by observing the changes in the cephalocaudal tumor cavity diameter after TSS in patients with pituitary adenoma. In our study, the preoperative cephalocaudal tumor cavity diameter in the DI group was higher than that in the non-DI group, which indicated that the degree of diaphragma sellar subsidence was greater in patients with high preoperative cephalocaudal tumor cavity diameter, and consequently, the risk of DI after surgery was also higher.

Nayak et al. showed that patients with pituitary adenoma presenting with visual abnormalities, having a suprasellar extension, or with large tumors were at a high risk of developing DI postoperatively [1]. They proposed that tumors that were large or had grown into the suprasellar region were closely associated with the pituitary stalk, and were prone to surgical disturbance, making the patients susceptible to DI after surgery. Nemergut et al. reported that patients with pituitary microadenoma needed greater surgical dissection to locate the lesions intraoperatively [3], and DI was also more likely to occur postoperatively in these patients. However, these studies only speculated that the risk of postoperative DI was due to injury to the hypothalamus–neurohypophyseal system, but did not provide any direct or indirect evidence supporting the claim. Our study showed that there was no difference in tumor size between the DI and the non-DI groups. However, we found that patients with giant pituitary adenoma were more likely to develop DI after surgery than those with macroadenoma. Woods et al. reported that patients with larger tumors in the suprasellar area, especially those with tumors extending toward the hypothalamus, had a greater risk of DI after TSS [23]. Patients with giant pituitary adenoma or tumors extending towards the hypothalamus have more pronounced diaphragm sellar sinking, which consequently stretches and damages the pituitary stalk and posterior pituitary. In this study, adenomas were classified into macroadenomas (1–4 cm) and giant adenomas (> 4 cm) according to the tumor diameter [24]. Although the size of giant adenomas is relatively large and greater changes in the craniocaudal diameter of the tumor cavity may occur after surgery, the latter can be tackled by intraoperative packing with gelatin sponge. From our experience, we recommend that the tumor cavity be filled as far as possible; however, the mass effect caused by excessive packing should be avoided. By the same token, the use of gelatin sponge limits the prediction of postoperative diabetes insipidus from tumor size.

Araujo-Castroet al. showed that total tumor resection was associated with a higher risk of DI [2]. They speculated that aggressive excision of large tumors was more likely to damage the remaining normal pituitary tissue or pituitary stalk, while retention of a large amount of tumor and pituitary tissue during conservative resection may preserve pituitary function. In addition, they only speculated upon the possible mechanism of damage as they did not look at subsidence of the diaphragm sellae.

The end point of tumor resection is reached when the entire diaphragma sellae folds down into the surgical field [19], indicating that the subsidence of diaphragma sellae may be significant when the tumor is completely resected, and insignificant otherwise. Therefore, we can infer that those patients with insignificant diaphragma sellae subsidence may have residual tumors, and as a result, the pituitary stalk is stretched lesser, and the risk of postoperative DI is lower. In our study, there was no correlation between the degree of tumor resection and the risk of postoperative DI, which may be because in patients with obvious subsidence of diaphragma sellae after total tumor resection, the tumor cavity was filled with gelatin sponge to reduce the degree of subsidence. Furthermore, the residual tumor in the cavernous sinus area may not affect the subsidence of diaphragma sellae. Ultimately, these confounding factors made it difficult to exactly predict the incidence of DI after surgery by the degree of tumor resection.

In addition to the tumor volume, cephalocaudal tumor diameter, and degree of tumor resection mentioned above, other reported predictors of DI after TSS include age and cerebrospinal fluid leakage [3, 17]. However, one cannot control these factors to reduce the risk of DI after surgery. Our study suggests that the change of cephalocaudal tumor cavity diameter (i.e., subsidence of diaphragma sellae) after TSS is an independent risk factor for DI. This factor can effectively help clinicians identify the population at risk of DI for postoperative management. Further, filling the tumor cavity with gelatin sponge may potentially reduce the change of cephalocaudal tumor cavity diameter, thus reducing the pull of the pituitary stalk, thereby mitigating the incidence of DI after surgery.

## Conclusion

In summary, our study is the first to investigate the change in cephalocaudal tumor cavity diameter before and after surgery on enhanced coronal sections of MRI. We found that the change in cephalocaudal tumor cavity diameter after surgery plays an important role in predicting postoperative DI onset in patients with a pituitary adenoma. Alterations in the diameter of the cephalocaudal tumor cavity reflect the subsidence of the diaphragma sellae, which can pull and damage the pituitary stalk, thereby leading to DI.

## Data Availability

The datasets generated and/or analyzed during the current study are not publicly available due to privacy or ethical restrictions, but are available from the corresponding author on reasonable request.
